# Applications of nanotechnology in smart textile industry: A critical review

**DOI:** 10.1016/j.jare.2022.01.008

**Published:** 2022-01-22

**Authors:** Mudasir Akbar Shah, Bilal Masood Pirzada, Gareth Price, Abel L. Shibiru, Ahsanulhaq Qurashi

**Affiliations:** a Department of Chemical Engineering, Kombolcha Institute of Technology, Wollo University, Ethiopia; b Department of Chemistry, Khalifa University of Science and Technology, Abu Dhabi 127788, United Arab Emirates

**Keywords:** Nanotechnology, Smart textile, Energy storage, Sensor, Nanogenerator, On-body electronics

## Abstract

**Background:**

In recent years, nanotechnology has been playing an important role in designing smart fabrics. Nanomaterials have been employed to introduce in a sustainable manner, antimicrobial, ultraviolet resistant, electrically conductive, optical, hydrophobic and flame-retardant properties into textiles and garments. Nanomaterial based smart devices are now also being integrated with the textiles so as to perform various functions such as energy harvesting and storage, sensing, drug release and optics. These advancements have found wide applications in the fashion industry and are being developed for wider use in defence, healthcare and on-body energy harnessing applications.

**Aim of review:**

The objective of this work is to provide an insight into the current trends of using nanotechnology in the modern textile industries and to inspire and anticipate further research in this field. This review provides an overview of the most current advances concerning on-body electronics research and the wonders which could be realized by nanomaterials in modern textiles in terms of total energy reliance on our clothes.

**Key scientific concepts of review:**

The work underlines the various methods and techniques for the functionalization of nanomaterials and their integration into textiles with an emphasis on cost-effectiveness, comfort, wearability, energy conversion efficiency and eco-sustainability. The most recent trends of developing various nanogenerators, supercapacitors and photoelectronic devices on the fabric are highlighted, with special emphasis on the efficiency and wearability of the textile. The potential nanotoxicity associated with the processed textiles due to the tendency of these nanomaterials to leach into the environment along with possible remediation measures are also discussed. Finally, the future outlook regarding progress in the integration of smart nano-devices on textile fabrics is provided.

## Highlights

• Current trends of using nanotechnology in textile industries.
• Nanotechnology-driven techniques for fabrication and modification of textile fibers.
• Wearable nanotechnology for energy storage, sensing, drug release, optics, electronics and photonics.
• Environmental concerns associated with nanotechnology processed textiles.

## Graphical abstract

**Figure:**
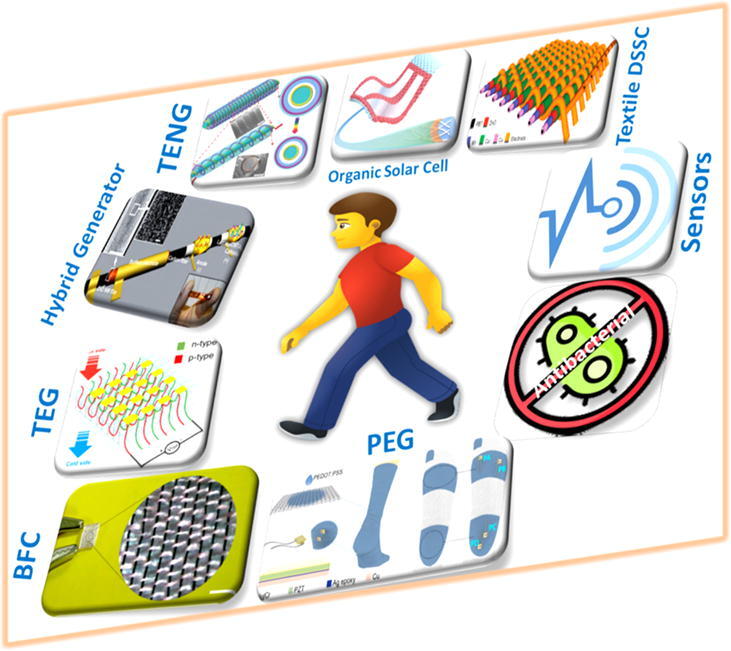

## Introduction

The modern textile industry faces incessant consumer demand for innovative applications of new technology and a constant stream of new and ever more innovative products. The ‘conventional’ textile industries have seen huge improvements in their products in terms of their mechanical strength and durability, the surface texture and ‘feel’ of the fabric and the ability to dye in a wide range of colours and printing patterns. Other developments include personal care factors such as anti-perspirant and deodourant properties along with flame-retardancy, self-cleaning and anti-microbial characteristics. However, recent years have seen the emergence of so-called ‘smart textiles’ which are derived from the combination of more conventional materials with smart nanomaterials. A smart textile is one which can sense changes in the environment and respond by modifying one or more of its parameters to perform a function [1]. There have been three generations in the development of smart textiles. First generation - or ‘passive’ - smart textiles are those that sense changes in the surroundings but cannot adjust their properties in response. For example, fabrics coated with various metal oxide nanoparticles can produce IR/UV resistant clothes; cotton impregnated with silver nanoparticles has anti-microbial properties. Second generation – or ‘active’ - smart textiles include fabrics which first percieve the changes or stimuli from the environment and then respond accordingly. Examples include thermochromic textiles which respond to changes in temperature by changing colour and shape-memory textiles which can respond to mechanical deformations. Third generation - also called ‘super-smart’ - active textiles are integrated with soft and smart electronics involving sensors, optical gadgets, nano-generators and energy storage devices. For instance, on-body electronics can offer sensing to various pollutants, diseases or threats. Also, attractive optical devices on a smart textile can be supported by nano-generators and energy storage devices [2], [3].

**Fig. 1:**
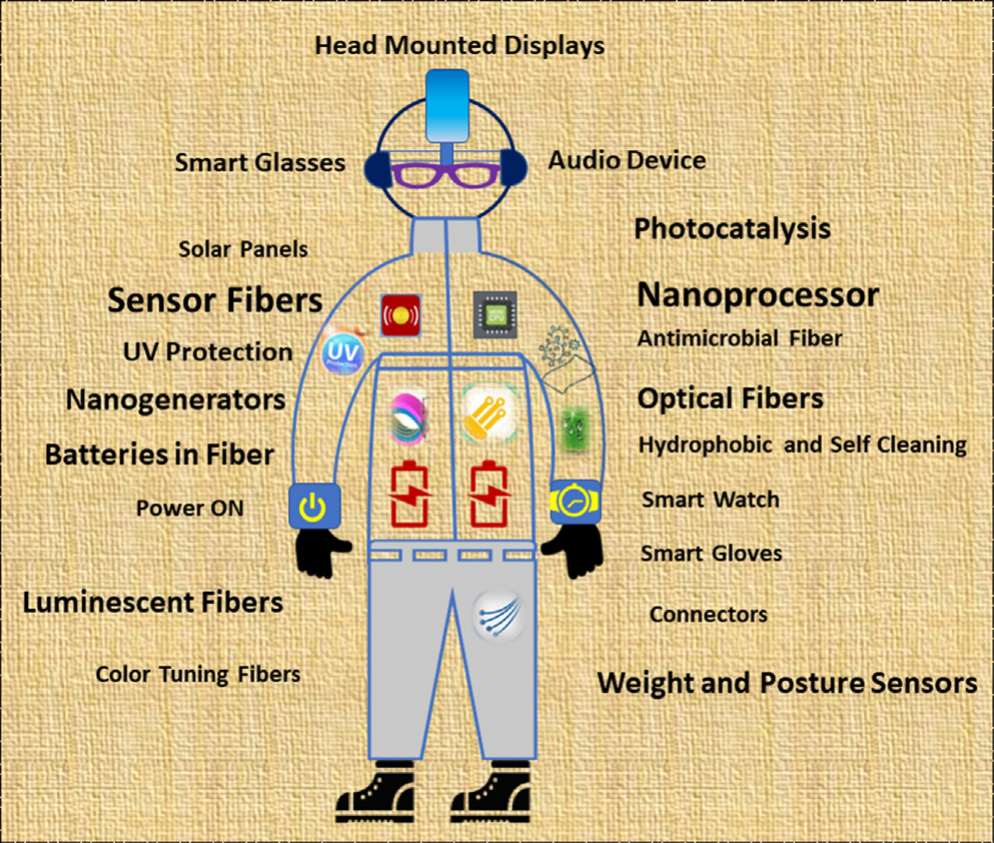
Outline illustration of futuristic smart clothing made from nanomaterial processed fibers for on-body multifunctional devices.

The incorporation of nanotechnology enables manufacture of smart and multi-functional textiles with many innovative applications in the areas of health, pharmaceuticals, fashion, sports, military, advanced protection and transportation (Fig. 1) [1], [2]. Connection to the ‘internet of things’ offers yet further potential for advanced uses. Fabrication of microelectronic devices is now at a level where they can be combined into textiles and allow the unique capabilities of nanomaterials to be exploited to add high added-value functionality to fabrics and garments while retaining other desirable properties such comfort, flexibility, lightness and aesthetic appearance [4], [6].

Many textile materials such as cotton, silk or polyester are ideal substrates on which to integrate smart, functional nanomaterials [3]. Various approaches have been developed to incorporate nanomaterials into textiles. The ‘bottom-up’ approach is used during the production of fibres from which the facrics are manufactured. By contrast, the ‘top-down’ approach is applied at the finishing stages, for example by printing technologies, spray coating, or impregnation. Electrospinning is a relatively new method for producing fibres and fabrics from processed raw materials and has been shown to be ideal for fabricating nanofibers [1], [4]. In coating technologies, various organic and inorganic compounds can be produced as particles in the nano-size range and can be directly utilised. Examples that have been used include polyacetylene, polypyrrole, polyaniline [5], Au [6], Ag [7], Pd [8], Cu [9], Si [10], CuO [11], ZnO [12], carbon nanotube (CNT) [13], [14], TiO_2_ [15], [16], chitosan [17], MXenes [18] and graphene oxide (GO) [19] nanoparticles. Textiles modified with these nanomaterials have potential applications in wound healing [23], [24], air purification [25], drug delivery [24], cosmetics, renewable energy generation and electronic applications such as fabrication of on-body diodes, transistors and circuitry [7].

The objective of this paper is to provide the reader with an overview of current and applications of nanotechnology in smart fabrics and to speculate as to potential future uses. The aim is to provide a comprehensive account of the latest advances in active and passive smart textiles as well as to give an insight to the latest research trends in modern textile industries. Possible environmental concerns associated with these novel textiles will also be highlighted. Hopefully, this will stimulate and inspire further research in this field.

## Current research trends for smart textile

In terms of ‘conventional’ textiles, modern fabrics have been developed that show high levels of performance with respect to hydrophobicity (wearer comfort), UV-resistance, antimicrobial, antistatic, anti-wrinkle, stain-free or shrink-resistance properties. However, these are ‘passive properties’ and researchers are interested in incorporating new fabrication and surface finishing methods to employ nanotechnology to inculcate smart and innovative applications. Their main motive is to introduce new applications with high efficiency without compromising on comfort, flexibility and light weight of the fabric. Table 1 summarizes some of the nanomaterials that have found application in this area.

**Table 1:** Summary of applications of smart textile integrated with various nanomaterials and nano-devices.

Functionality	Textile Substrate	Synthesis Method	Integration Method	Nanomaterial	Applications	Ref
Antimicrobial	Cotton	Sonochemical	In-situ deposition	Ag nanoparticles	Antimicrobial, anti-fouling	[270]
Antimicrobial	Cotton	Sonoenzymatic	Sonochemical deposition	ZnO/Gallic Acid	Biocompatible and antimicrobial fabrics	[54]
Antibacterial/Dye Degradation	Cotton	Hydrothermal/Sol- gel	Impregnation method	TiO_2_	Self-cleaning textile	[271]
Photo-degradation/Self-cleaning	Cotton	Sol-gel	Photo-deposition	Au/TiO_2_ film	Self-cleaning textile	[49]
Self- cleaning	Polyester fabric	Micro emulsion Water-in-oil	Silks screen printing	Copolymer/SiO_2_ nanocomposite	Textile coloration	[272]
Super-hydrophobicity	Cotton	Emulsion	Spray coatings	SiO_2_ nanoparticles	Absorbed in Oil-water interfaces	[273]
Super-hydrophobicity	Cotton	Sol-gel	Sol-gel	Perfluorooctylated quaternary ammonium silane /SiO_2_	Oil Repellency	[68]
Super-hydrophobicity	poly-(ethylene terephthalate) (PET)	Chemical deposition	Chemical deposition	Janus SiO_2_	Water-repellent textiles	[69]
Hydrophobicity and Insulation	Polyester-woven fabric	Fluorocarbon finishing	Electro-spraying	Silica aerogel	Hydrophobic and Heat insulating textiles	[274]
UV-Resistant	Cotton	In-situ polymerization	-----	PANI/TiO_2_	UV Protective clothes	[82]
UV-Resistant	Cotton	Acid extraction/Sol-gel	Spray coating	polyurethane based MnO_2_-FeTiO_3_	UV Protective clothes	[83]
Fire retardancy	Wool fabric	Precipitation	Pad batch	Nano-kaolinite	Fire proof textile	[275]
Antistatic Properties	Polyethylene therephthalate/Cotton	Acid hydrolysis	Dip Coating	Aminoalkyltrialkoxysilanes	Textile finishing	[86]
Antistatic Properties/Breathability/Moisture-Wicking	Polyacrylonitrile	Electrospinning	-----	β-Cyclodextrin/Polyacrylonitrile	wearing comfortability in textile	[276]
Antistatic Properties	Polyethylene terephthalate	Melt-spinning	-----	Carbon black/Polypropylene/polyamide (Nylon)	Antistatic textile	[277]
High conductivity	Nanofiber	Chemical method	Electro-spraying	Mn@ZnO/CNF	Energy storage on textile	[73]
High conductivity	Polyacrylonitrile nanofiber	Electro-spinning	-----	Graphene oxide	Wearable electronic devices on textile	[278]
Supercapacitors	Stainless steel fibers	Twist-bundle-drawing technique	-----	PPy@MnO_2_@rGO@Conductive Yarns	Energy Storage on textile	[103]
Supercapacitors	Stainless steel yarn	Microwave-assisted hydrothermal method	-----	Fe_3_O_4_/PPy	Self-healing textile fibers for energy storage	[104]
Battery	Al and Cu based fibers	Fiber drawing method	-----	Al–NaOCl galvanic cells	Energy Storage on textile	[115]
Battery	poly ethylene oxide	Drawing/Extrusion method	-----	LiFePO_4_ (cathode)/Li_4_Ti_5_O_10_ (anode)/solid poly ethylene oxide (electrolyte)/PVDF	Flexible Energy Storage fibers for textile	[279]
Light Emitting Diodes (LEDs)	Soft Fabric	lamination and spin-coating	-----	Polyurethane/poly(vinyl alcohol) (PVA) layers	Lighting effect on textile	[117]
Light Emitting Diodes (LEDs)	polyester	surface-replicating method	-----	Poly-vinyl alcohol/SU-8 (planarization layer)/Si-base elastomeric (strain buffer)	Clothing-type displays	[280]
Photonics	gold-coated fabric	single-pulse laser ablation technique	-----	Au nanoparticles	Printable holography on textiles	[281]
Photonics	multi-walled carbon nanotube sheets	Chemical vapor deposition	Anchoring	MWCNT/Fluorescent dyes	Fluorescent supercapacitor fibers	[126]
Photonics	computerized Jacquard loom	-----	layer-by-layer deposition	polymer photonic bandgap (PBG) fibers	smart cloths, signage and art	[282]
Photonics	Silicone fibers	Extrusion	Warp and weft weaving	Geniomer 200 (polysiloxane-urea-copolymer with a polysiloxane)	Pressure sensor based flexible optical fibers for textiles	[283]
Biomedical	Cotton fabric	Anionic exchange method	Impregnation	NanoTiO_2_@DNA	Delivery of drugs based on nanomedicine	[284]
Biomedical	grooved solid and hollow hydrogel fibers	3D-printing and casting	weaving, braiding, and embroidering	polylactic acid/polydimethylsiloxane (PDMS)	Tissue engineering;wearable or implantable medical devices; and soft robotics	[285]
TENGs	PTFE film	Sputtering/Etching	Sputtering method or simple adhesion	PTFE/Cu film	Sustainable wearable or portable electronics and smart sensor networks	[203]
TENGs	spring and elastomer	-----	Laser cutting and gluing	Acrylic sheets/spring/Silicone/Carbon nanofiber	Harvesting and sensing of vibrational energy, such as from vehicle, building, waves, wind, walking, etc.	[204]
PENGs	Silicone Rubber	freeze-drying method	doctor-blade deposition	Bi_1−x_Sm_x_Fe_1−x_Ti_x_O_3_ /Cellulose	self-powered mechanosensation system[	[222]
PENGs	micropatterned P(VDF-TrFE) polymers	Photolithography process	Spin Coating	poly(vinylidenefluoride-co-trifluoroethylene) (P(VDF-TrFE)	Vibrational sensor/weather sensor	[223]
BFCs	CNT yarn	vapour-phase polymerization	Biscrolling	MWCNT/PEDOT/Glucose oxidase	On-body glucose energy harvesting	[286]
BFCs	Metallic Cotton Fibers	-----	Layer-by-layer assembly	poly(ethylenimine)/(tetraoctylammonium bromide-stabilized Au nanoparticle (TOA-Au NP)/tris-(2-aminoethyl)amine (TREN))_n_, m-GO_x_: GO_x_/tris-(2-aminoethyl)amine (TREN)_m_	On-body glucose energy harvesting/Sensing	[251]

Functionalities explored over the past couple of decades include bacterial resistance [20], ease of dyeing [21], hydrophobicity [22], flame retardancy [3], UV protection [23], colorfastness [24] and ability for self-cleaning [25]. As described below, an area of intense current activity is to develop fabrics, and hence garments, that can harness, store and deliver energy to the wearer to power daily activities. Possibilities include the incorporation of piezoelectric generators, solar cells or biofuel cell modules. In this regard, various kinds of nanogenerators [26] and supercapacitors [27] have been designed and integrated with the textile. Researchers have also developed devices which can sense external stimuli and generate electronic signals for various monitoring systems [28].

Many of these innovations began in designs for the fashion industry as reviewed by Yetisen et al. in reference [3]. In this direction, Philips has designed a range of clothing (Bubelle) which shows change in colour on the basis of the disposition of the wearer. Black Eyed Peas developed the fashion clothings on the basis of Organic Light Emitting Diode (OLED) materials so as to decorate the wearer with range of lighting patterns and colour effects [3]. Fashion industry also featured three dimensional (3D)-printed bubble machine dress (Anemone), Parametric Sculpture Dress, Cipher-a cloth embroided with animated black mirrors, and a flying drone like dress called ‘Volantis’ using advanced nanomaterials [3]. Researchers focus on exploring nanomaterial based photonics over the textile so that highly aesthetic, colourful and smart clothes, which can change the colour of the clothe as per various stimuli, can be developed.

Although there have been a number of advances in this field, there remain limitations and challenges which hold back the smooth progess of this industry. The main challenges lie in the integration process as the fabricated devices and on-body electronics often spoil the smoothness, appearance, comfort and wearability of the clothes. Moreover, the efficiency and durability of the on-body devices and electronics needs significant improvement so that completely self-reliant clothes can be realized. As well as loss of performance, leaching and loss of the nanomaterials raises environmental issues as concerns persist regarding potential nanotoxicity.

A number of categories of smart textiles will now be discussed in detail.

## Antimicrobial textiles

The antibacterial guard to the textile is very interesting and beneficial for human health. Various antimicrobial agents such as TiO_2_ [29], chitosan [30], N-Halamine [31], Ag [32] Cu_2_O [33] and metal/hemp fibers [34] etc. have been incorporated into fabrics for antimicrobial activities [35], [36], [37]. To develop an antimicrobial textile, the active nano-materials can be incorporated chemically or physically into the fabrics [38]. Muñoz-Bonilla and Fernández-García [39] developed antimicrobial nano-materials by using various methodologies, such as electrospinning, nano-precipitation and self-assembly. They investigated the surface of nanostructured polymeric films and their antimicrobial behavior.

Among the most primitive antimicrobial nanoparticles used over textile surfaces is silver (Ag). It acts as a doping antimicrobial agent, and reveals to have outstanding antimicrobial activity without changing its mechanical properties [11]. Ag nanoparticles being very small in size, contains a very high surface area which eventually helps them to interact with bacterial proteins and inhibits their cell growth. Ag nanoparticles also interfere with the electron and substrate transport system [40]. The Ag^+^ ions produced on reacting with moisture, diffuse fast across the cell wall and cell membrane and reach into the cytoplasm. On the cell membrane, the Ag^+^ ions react with the S-containing proteins and alter the cell wall morphology [41]. As a result, the cell membrane gets deteriorated and releases the cytoplasm due to osmotic action. The Ag^+^ ions also interact with the phosphate containing proteins to condense DNA, which eventually causes cell death [42]. The extent of antimicrobial action by Ag nanoparticles is the function of size, surface area, concentration and production of Ag^+^ ions [43]. Patil and co-workers [44] investigated fast one step sono-chemical synthesis and deposition method to obtain silver coated cotton nanoparticles. They revealed that the silver nanoparticles were stable, mono-dispersed, uniformly deposited on the cotton fabrics and exhibited highest antimicrobial activity. Ag doped SiO_2_ nanoparticles with core − corona morphology were also explored for antibacterial and self cleaning function on cotton fabrics [45]. These corona-structured nanoparticles can be made graves for the bacteria by loading antibacterial compounds such as quaternary ammonium salts on these structures [46]. TiO_2_ can produce reactive oxygen species (ROS) such as superoxide, hydroxyl radical or a positive hole [47]. These ROS can interact with the cell wall and cell membrane of the bacteria and eventually lead to cell death. This property of TiO_2_ nanoparticles have been exploited in antibacterial textiles [48]. The ROS can also decompose the organic matter or oily dirt and hence can impart self-cleaning property to textiles. This self-cleaning property can be further enhanced if TiO_2_ is doped with some other active species like Ag, Au or SiO_2_, ZnO etc. [49]. Riaz and co-workers [50] investigated the applications of TiO_2_ with 3-(trimethoxysilyl) propyl N,N,N-dimethyloctadecylammonium chloride and 3-glycidoxypropyltrimethoxysilane in textiles industry. They concluded that treated cotton showed durable super-hydrophobicity, self-cleaning and antibacterial activity. ZnO nanoparticles also behave like TiO_2_ and exhibit antibacterial and self cleaning properties for textiles loaded with Gram-negative Escherichia coli and aerobic Gram positive Staphylococcus aureus. Patil and co-workers [51] investigated sono-chemical synthesis processes for production of ZnO nanoparticles and its incorporation on cotton fabrics. The ZnO nanoparticles finished upon cotton fabrics exhibited flexural rigidity, tensile strength, water contact angle and air permeability. They showed excellent deposition properties of the nanoparticles on cotton fabric yarns along with significant antibacterial properties. Fouda and co-workers [52] combined bio-active macromolecules secreted by bio-synthesized ZnO and fungi nanoparticles for antibacterial activity and UV protection. They extracted proteins that have an affinity to cap ZnO nanoparticles using an isolated fungus, Aspergillus terreus. They revealed that biosynthesized ZnO nanoparticle coated on textile could inhibit pathogenic bacterial growth with respect to the untreated fabrics. Karthik and co-workers [53] employed green synthesis to make ZnO nanoparticles which showed significant antibacterial action. Salat and co-workers [54] also carried out coating of cotton medical textiles with gallic acid and antibacterial ZnO nanoparticles. They demonstrated that gallic acid provides a safe contact of the coated materials with the antibacterial agent, cross-linked phenolic network and human skin. Hiremath and co-workers [55] developed magnetite nanoparticles using green synthesis with the help of ultrasonication method which exhibits effective microbe protection. Yu and co-workers [56] fabricated nano-fiber core-spun yarn with a highly efficient antibacterial properties with the help of electrospinning. The yarn structure possesses almost 100% antibacterial characteristics.

Nanomaterial processed face masks have been in wide focus since the outbreak of COVID-19. Various researchers developed antiviral face masks and Personal Protective Equipment (PPE) kits which could filter various pathogens including SARS-CoV-2. Talebian and co-workers (2020) proposed two methods to control COVID-19 involving nanomaterial based disinfectants and biosensors, respectively on mask or PPE fabrics. They suggest that metallic nanoparticles such as Ag, Cu, TiO_2_ etc. can be alternatives to the traditional disinfectants viz; chlorides, quaternary amines, peroxides, and alcohols; owing to their excellent antiviral activities. They also propose that highly efficient biosensors can be integrated on face mask or PPE kits so that early detection of SARS-CoV-2 or other viruses can be realized [57]. Lustig and co-workers (2020) developed multi-layer face masks containing alternate hydrophilic and hydrophobic layers. They found that the hydrophobic layer repels the aqueous aerosol on the hydrophilic layer which inhibits the wicking movement. These face masks are proposed to prevent spread of virus via sneezing and coughing [58]. El-Atab and co-workers (2020) prepared a nanoporous and flexible Si-based template on which a flexible and lightweight polymeric membrane was developed. The membrane was attached on a reusable N95 mask which could filter microbes upto the size of 5 nm [59]. Thus, various nanomaterial combinations can be integrated with the textile fibers by drawing them into nanofibers or by coating methods, so that optimum activity can be obtained [60], [61], [62], [63].

## Hydrophobicity and oleophobicity in textiles

Nature is the true designer of smart functional materials. It has often inspired the researchers to mimic the biological phenomena. Same thing can be observed in case of hydrophobicity phenomenon. For example, the ducks are bestowed with preening oil coated feathers; which helps them to survive in water. The researchers mimic this natural phenomenon by using chitosan coatings over cotton and polyester textiles. The chitosan coating solution was developed by a precipitation method; which was further processed using a silicone compound so as to obtain lower surface energy [64]. Similarly, the researchers employed pristine and surface modified carbon nanotubes (CNTs) on the cotton fibers so as to mimic the surface texture of lotus leaves (Lotus effect) to produce superhydrophobic surfaces [65]. A large contact angle of more than 150° was obtained. One more such work was done by Ramaratnam and co-workers [66] which involved the development of hydrophobic nanocoatings (20 nm) so as to achieve hydrophobic fabrics. Water repellent fibers can be also developed by using hydrocarbon mounted nanowhiskers. These materials have dimensions of the order of one-third to that of conventional cotton fibers. These nanowhiskers can be introduced in the textile fiber so as to achieve kind of peach fuzz effect. The distance among the individual nanowhiskers is less than the size of a water drop but more than the molecular size of H_2_O. As a result, significant surface tension can be realized which doesn’t allow water to spread on its surface. However, the breathability can be maintained owing to the permeability of nanowhiskers. Hence, water repellent coatings can be developed by nanoparticulate films on the textiles. Fluorinated mixtures are being regularly used for this application on textile polymers [67]. Using proper processing method for tuning the texture of fibers, superhydrophobicity can be attained without deteriorating the comfort, softness and durability of the fabrics. Tuning of contact angle is instrumental in attaining the hydrophobicity or oleophobicity. A significant contact angle of more than 130° was achieved when SiO_2_ nanoparticles (143–378 nm) were used along with a water repelling agent. SiO_2_ nanoparticles can also be used along with perfluorooctylated quaternary ammonium silane (PQAS) as the coupling agent [68]. A nice contact angle of 145° was obtained which lead to excellent hydrophobicity, owing to the diminishing of surface energy by PQAS. The oleophobicity was also enhanced; exhibiting contact angle of 131° when a droplet of diiodomethane (CH_2_I_2_) was used on the cloth surface. Amphiphilic Janus type micro/nanoparticles were also mounted on the textile surfaces to achieve hydrophobicity [69]. The microparticles help in crosslinking between the fibers, while the nanoparticles stuck to the surface of fiber.

SiO_2_ nanoparticles along with an epoxy-containing poly(glycidyl methacrylate) (PGMA) was used for making a primary nanocoating layer on the fabric surface which was then further processed by different functional polymers containing amino, anhydrido, carboxy, and hydroxyl functional groups [66]. The researchers are trying to impart both the hydrophobic and oleophobic properties to textiles. For example, SiO_2_ nanoparticles were used on cotton fabrics followed by hydrophobization with poly(dimethylsiloxane) (PDMS). As a result, a nice contact angle of 155° was obtained for a water droplet [70]. They further introduced the oleophobicity in it by treating it with a perfluoroalkyl chain. Using oil droplets, a static contact angle of 140° and a roll-off angle of 24° was obtained. The various primary applications considering hydrophobicity/oleophobicity are waterproofing [71], anti-fouling [72], controlled wettability [73], self-cleaning [74], water repellency [75], oil/water separation [76], anti-icing [77], and anti-corrosion [78].

## Ultraviolet-resistant textiles

The UV protection materials are obtained by treatment of fabrics with UV-blocking (UVB and UVA radiations) nano-materials so as to improve the UV shielding. The UV protection efficiency is measured by ultraviolet protection factor (UPF) and depends on the nature of the fabric.

The nanomaterials responsive to UV light such as TiO_2_ and ZnO are capable to scatter or absorb UV radiations [47]. These materials are stable and non-toxic and can be stable even at higher temperatures. The scattering of UV light by the nanoparticles is a function of nanoparticle size and wavelength of the radiation. TiO_2_ nanoparticles have been used on cotton as the UV blockers. The durability of the TiO_2_ finishing was found to be good even after 50 washings [79]. ZnO nanorods have also been used as the efficient UV scattering layer on the cotton fabric [80]. Furthermore, ZnO nanoparticles have been applied on cotton and polyester fabrics as UV absorbing layer [81]. Yu and co-workers confirm the anti-UV properties of polyaniline/titanium dioxide (PANI/TiO_2_) and polyaniline (PANI) cotton fabrics [82]. Dhineshbabu and Bose endorsed that combination of MnO_2_-FeTiO_3_ nanoparticles with thermoplastic polyurethane cotton textiles helps to block UV rays [83]. The results confirm that nano-coated materials on the textile fabrics possess strong UV-blocking capacity, an intelligent and durable fabric as compared to uncoated materials. UV-absorbing phenomena is of great application in textiles as it can be useful in protecting the humans from harmful UV exposure.

## Antistatic properties in textiles

Nylon and polyester being hydrophobic exhibits larger static charge. Contrary to this, the cellulosic fibers have higher moisture which decreases their static charges. Various nanomaterials have been employed to achieve antistatic properties in synthetic fibers viz; ZnO whiskers [84], TiO_2_ nanoparticles, Sb-doped SnO_2_ nanoparticles etc. These nanomaterials dissipate the static charge on the textile due to their conductive nature. Some nanosols based on silanes have also been used as antistatic agents as they absorb moisture from air by interacting through its surface hydroxyl groups. Commercially, poly(tetrafluoroethylene) (PTFE) antistatic membrane was developed which has conductive nanoparticles attached to the membrane [85]. Some researchers developed sol − gel coatings on the surface of the fiber to achieve antistatic properties [86]. Various hydrophobic chemical species such as alkoxysilanes are also employed after modifying it with hydrophilic compounds or amino group containing alkoxysilanes. Sol − gel-coated textiles exhibit antistatic properties as they contain hydrophobicity on the surface but moisture deep under the coatings. Silver nanoparticles with fluorine hydrophobic finish can achieve antistatic properties in polyester fabric [87]. ZnO nanoparticle coatings have also been reported to show antistatic characterictics [88]. The silver nanoparticles could decrease the static voltage of polyester fiber by 60.4%. Whereas, when Au, and ZnO nanoparticles were combined, the decrease in the static voltage was by 77.7%. One more study reported Sb-doped SnO_2_ for antistatic properties in polyacrylonitrile (PAN) fibers [89]. These nanoparticles when diffused into the fibers generated conductive channels, which eventually lead to antistatic characteristics.

## Electrically conductive textiles

Introduction of sensors and actuators in the textile industry is mainly pivoted on the conductive properties of the textile material. Conducting polymers find a vast application in this regard in textile industry. The tuning of resistivity in these materials produces electric response on textile surface when it is exposed to an external stimulus. These polymers can be modified to a desired property by incorporating a variety of nanomaterials into its matrix. For example, nanostructured polyaniline (PANI), polypyrrole (PPy) and polythiophene (PT) are the widely used conducting polymers which can impart enhanced mechanical strength, optical and conducting characteristics. These polymers have many advantageous features for integration with the textile industry viz; lower production costs, flexibility and light weight.

Many conductive nanomaterials have been introduced to modify the surface structure of the fibers so that various smart functionalities can be achieved. Surface processing of fibers by conductive polymers enhances their conductivity by magnitude of one order [90]. For example, SiO_2_ nanoparticles have been blended with polyimidoamide fibers using a spinning method. Electrically conductive channels have been developed in the fibers when nanoparticles were introduced in polyacrylonitrile (PAN) fibers. This lead to increased antistatic and mechanical features [89]. SiO_2_ nanoparticles along with diamine (diaminodiphenylmethane) and montmorillonite have been used to coat the fibers to enhance their tenacity and thermal resistance [91]. Conductive polymers such as PANI, PPy and PT can be used to inculcate enhanced tensile strength and thermal stability in the synthetic fiber by employing chemical oxidative deposition. These composite fibers can find wide applications in electromagnetic shielding, microwave attenuation and reduction of static electrical charge. Many conductive matrices have been developed for coating cotton to impart electrical conductivity. Shim and co-workers [92] developed a polyelectrolyte-based coating mixed with multiwalled carbon nanotubes for conducting textiles. Mattana and co-workers [93] used a blending mixtures of different metal nanoparticles conformally mounted around the heterogeneous contour of cotton fibers. The mechanical deformations in the cotton based transistors can be mitigated by introducing in-situ polymerization as it leads to the formation of flexible bridges between the nanoparticles. Graphene has also been introduced in textile fibers to inculcate the conductive features. For example, two sets of graphene microribbons were interlaced to prepare a fabric [94]. The as-prepared fabrics exhibited good durability. The conductivity of this fabric was tuned and optimized by changing the density of packing ribbon. Atmospheric chemical vapor deposition (CVD) was employed to generate graphene fibers while using Cu meshes as the substrate which contained wires of ∼60 μm in diameter. Similarly, graphene can be immobilized on a fabrics using conventional dip and dry methods. In this method graphene oxide is reduced to graphene and multilayers are produced which enhance the fabric conductivity upto 3 folds [95]. This surface conductivity can be tuned by choosing a proper reducing agent and its concentration. In this case, an electrical resistivity of 103 to 106 kΩ-cm^−1^ was achieved in the graphene coated cotton fabric [95]. Trovato and co-workers [96] developed a versatile and new method to achieve a dispersion in water-based paste of short sized carbon nanotubes (CNT) for the production of electro-conductive textiles. They showed nanotubes are well dispersed on coatings and fabricate wearable conductive materials. This suggests that various conducting 2D and 3D nanomaterials which can be drawn into wires and films; or coated on the textile fibers, can find multiple on-body electronic applications.

## Energy storage by textiles

Supercapacitor for energy storage applications have been applied in the textile technology. Researchers are looking for introducing supercapacitor electrodes into the fabric without disturbing the flexibility and wearable characteristic of the fabric [97]. Cotton and polyester fabrics have been modified using activated carbon in poly(methyl methacrylate) (PMMA) and polyethylene glycol (PEG). Screen printing was adopted on polyester microfibers so as to arrange the supercapacitor cells in a conventional symmetrical two-electrode setup. The activated carbon coated electrodes on cotton/polyester exhibited a gravimetric and areal capacitance of 85 Fg^−1^ at 0.25 Ag^−1^ [97]. Recently, Zhou and co-workers (2021) [98] prepared in situ cross-linked polyvinyl alcohol/phase (PVA/PCM) nano-fiber materials using an emulsion-electrospinning process. They revealed that, PVA/PCM nano-fibers possess excellent durability, thermal stability, energy storage, improved water resistance and tensile strength that leads to significant applications in heat storage and temperature regulation as compared to the normal PVA/PCM nano-fibers. Lai and co-workers [99] analyzed a new strategy to synthesize the wire-shaped solid-state supercapacitors using a soft aerogel in a facile dip-coating process. They electro-spunned polyacrylonitrile nano-fibers hydrophilically using glycerol on titanium metal wire to form the sacrificial aerogel with a huge void volume. They showed that the capillary effect in the natural drying process can slow dissolution of template in the solvent, and the polystyrene-sulfonate (PSS) etching may lead to a mesoporous morphology. They concluded Ti/poly (3,4-ethylenedioxythiophene) (PEDOT) is a very powerful source for wearable electronics. Pan and co-workers [100] developed a flexible supercapacitor mounted textile by using CNT/PANI composite fiber [100]. These supercapacitor textiles could do photoelectric conversion and store energy in a stacked multilayer structure. The carbon nanotubes have been developed by CVD which was then woven into the fibers by first making a thicker film by stacking. The as developed textile fiber was electrodeposited with PANI to form an electrode. A gel electrolyte was used to coat the electrode so that a supercapacitor is created. This material exhibited a capacitance of 272 F-g^−1^ with maintenance of 96% even after 200 bending cycles [100]. Zhang and co-workers [101] looked to enhance the performance of the textile by designing supercapacitors where a metal wire is kept at the centre of the carbon nanotube yarn. A core or sheath shaped carbon nanotubes yarn is formed by one-step continuous spinning which lead to the formation of linear supercapacitors.

**Fig. 2:**
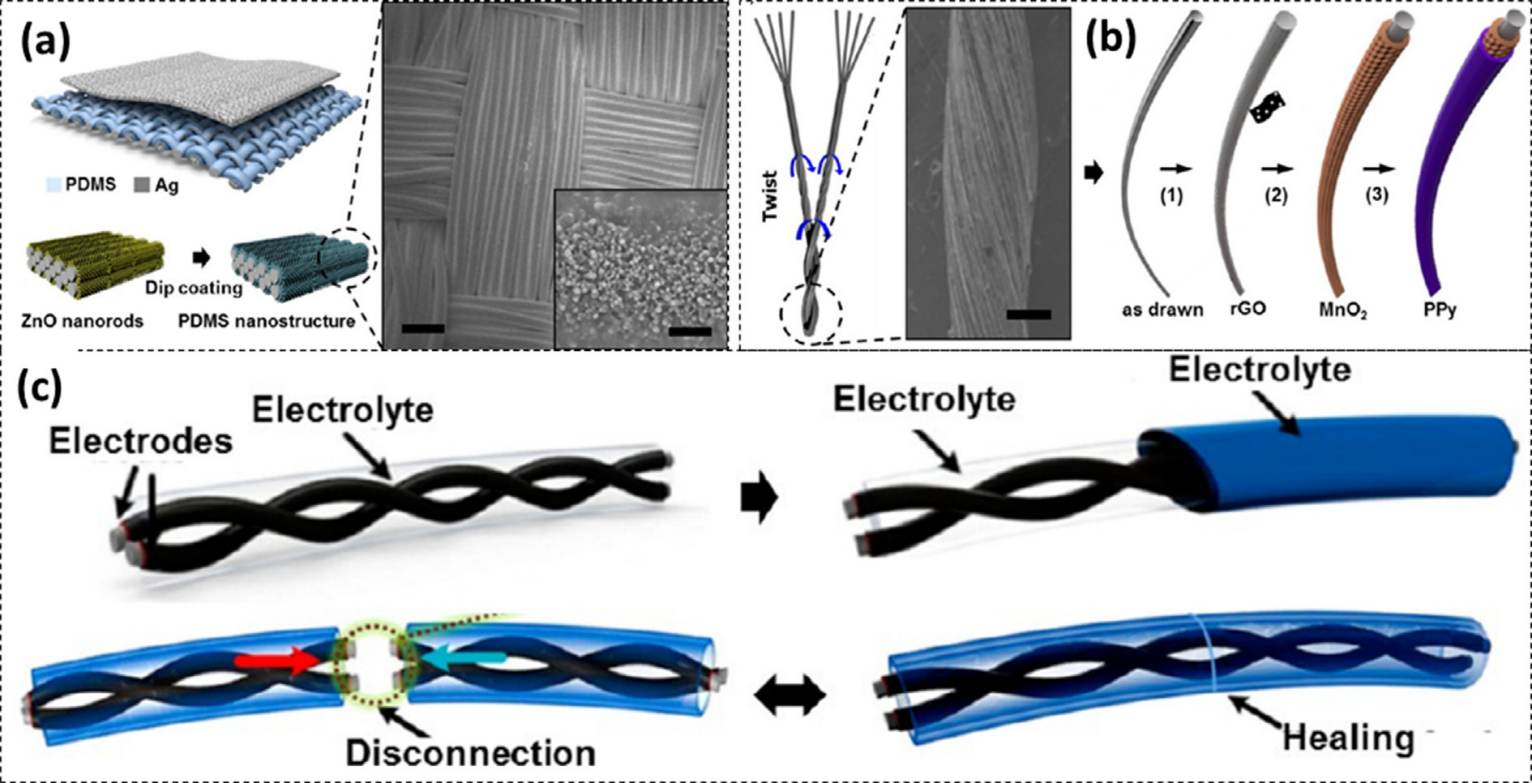
Power production in textile: (a) Development of a piezoelectric hybrid nanogenerator from a nanopatterned TENG where PDMS nanopatterns are being templated on ZnO nanorods (Inset: SEM micrographs of ZnO nanowires used) Reproduced with permission from Ref. [102] Copyright 2015 American Chemical Society (b) Fabrication of yarn from the nanofibers functionalized with PPy, rGO and MnO_2_. Reproduced with permission from Ref. [103] Copyright 2015 American Chemical Society (c) Fabrication of supercapacitors from self healable yarn. Reproduced with permission from Ref. [104] Copyright 2015 American Chemical Society.

Triboelectric nanogenerators are smart energy efficient devices have also been developed on wearable textile [102]. The fabrication of a device involving nanopatterned PDMS structure has been presented in Fig. 2 [3]. The polydimethylsiloxane (PDMS) nanopatterns developed over ZnO nanorod arrays have also been exploited for the development of triboelectric nanogenerators. These devices exhibit output voltage of 120 V at 65 μA, whereas, its four-layered structure could generate output voltage of 170 V at 120 μA. There was an insignificant drift even after 120,000 cycles which indicated their stability [102].

Kim and co-workers [105] activated the cotton fibers by coating with carbon material for developing a energy producing textile. These processed textiles could generate electrostatic energy frictional stimulations. An open-circuit voltage of − 60.9 V could be obtained from these materials [105]. Some researchers designed nanogenerators in textile by exploiting piezoelectricity along with electrostatic forces [106]. The piezoelectric and electrostatic effects could be hybridized when ZnO nanowires were used in the textile fiber along with discharge films (Fig. 2b). An output voltage of 8 V was obtained at 2.5 μA by this nanogenerator. This power source was capable to work in liquid crystal displays (LCDs) and OLEDs [106]. Twist-bundle-drawing was a new technique used to produce pristine soft conductive yarns [103] (Fig. 2c). When processed PPy, MnO_2_ nanosheets or reduced graphene oxide (rGO), weavable supercapacitors could be produced. Huang and co-workers [107] developed stretchable supercapacitors based on PPy by electrodepositing PPy on stretchable stainless steel meshes.

One of the challenging aspect of this technology is that the fixing of broken yarn electrode is quite difficult [104]. Hence, the fibers with supercapacitors could have self-healing characteristics. These self healing electrodes were developed by wrapping magnetic electrodes around a self-healing polymer shell. The broken fibers are actually rejoined by the magnetic attraction so as to restore electrical conductivity, while the configurational integrity is maintained by the polymer shell. The cotton yarns are coated with PEDOT − poly(styrenesulfonate) nanolayers which are based on an array of Au nanoparticles. These nanolayers made the cotton yarns conductive and could transfer solar energy along the whole dress [108]. The new trend in the designing of smart textiles is the development of multifunctional nanocomposite fibers. These structures can find applications in fiber optics and batteries in the textile. These fiber nanocomposites can be further processed while drawing, using different biofunctional polymers [109], [110], optical plastics [111], [112], conductive polymers [113], metal alloys [114], and electrochemical materials [115].

Flexible fiber batteries can result in promising functional textiles. Various flexible fiber batteries have been made using simple inorganic materials [115] or Li-ion [116]. A simple such fiber battery was made using a microstructured low-density polyethylene (LDPE) jacket containing a channel network all along the fiber. A typical Al/air galvanic cell was designed inside a fiber when a double strand of Aluminum (Al) and Cu wires acted as anode and cathode, respectively. The spacings between the two were filled with an electrolyte such as sodium hypochlorite (NaOCl). The advantage with the fiber based Li-ion battery is that it can be cut into stripes and can be used directly in the textile. Two prototypes of textiles have been proposed using these stripe batteries decorated fibers. In one such prototype, a wool textile matrix was taken and fiber batteries were incorporated [115]. This fiber could lit up an LED and also a wireless mouse could be operated [115]. Another prototype involved flexible stripe Li-ion batteries [116]. The stripe batteries based flexible fiber is considered as a nice method to mount wearable power generation entities on textile. These materials could be of great use to supply power to the LEDs and other nanogenerators during the fashion shows.

Organic Light Emitting Diodes (OLEDs) have been extensively introduced in soft fiber to produce smart wearable fabrics [117]. Schottky diodes have also been introduced on fabrics. The Schottky diode have been synthesized by employing a photoresist and reactive plasma ion etching of the ZnO nanorods [118]. Textiles decorated with Schottky diodes can find nice applications in voltage clamping, switched-mode power supplies, and reverse current and discharge protection. Polymer yarns twisted with metal wires have been utilized for the development of electromagnetic shield fabrics. A bismuth − tin (Bi_42_Sn_58_) based polycarbonate cable have been designed using stack-and draw method [114]. In this method, a molten Bi_42_Sn_58_ alloy was filled into a polycarbonate tube and was then drawn into a cable. Indium [119] or tin − zinc [120], [121] based polymer or wire nanocomposites have been developed using the same drawing method. These polymer/metal wire composites have found applications in designing advanced materials and optical devices along with electromagnetic shielding.

## Photonics in textiles

The use of photonic technologies in the fashion industry attracted a vast attention. The various optical materials viz; optical fibers, optical films and nanoparticles have been employed to design various attractive and smart textile fabrics. The objective behind the use of photonic material in textile is to tune the appearance of the dress by modifying the pattern of light and colour intensity. For example, the optical films developed from periodical dielectric multilayers can be robustly coated on the textile fibers which could result in highly reflective and colourful designs on the fabric when observed at varying angles. Holographic film coatings can also be applied on fabrics to generate attractive 3D visual effects [122]. Phosphorescent films have also been employed on fabrics to make it glow even in the dark [123]. Photochromic and thermochromic materials have been used in textile so as to sense change in temperature or intensity of light [124]. Retro-reflective inks on textile have been extensively used for security clothing. Electroluminescent wires [125], fluorescent fibers [126], optical fibers [127] and photonic band gap fibers [128] have also been found to have advanced functions in smart textiles. Apart from the aesthetics features in fashion industry, these nano-processed fibers can perform various other functions such as temperature sensing [129], humidity sensing [130], pressure [131], strain [132], optical displays [133], data transfer and communication in advanced textile [134].

### Color-tunable optical fibers

**Fig. 3:**
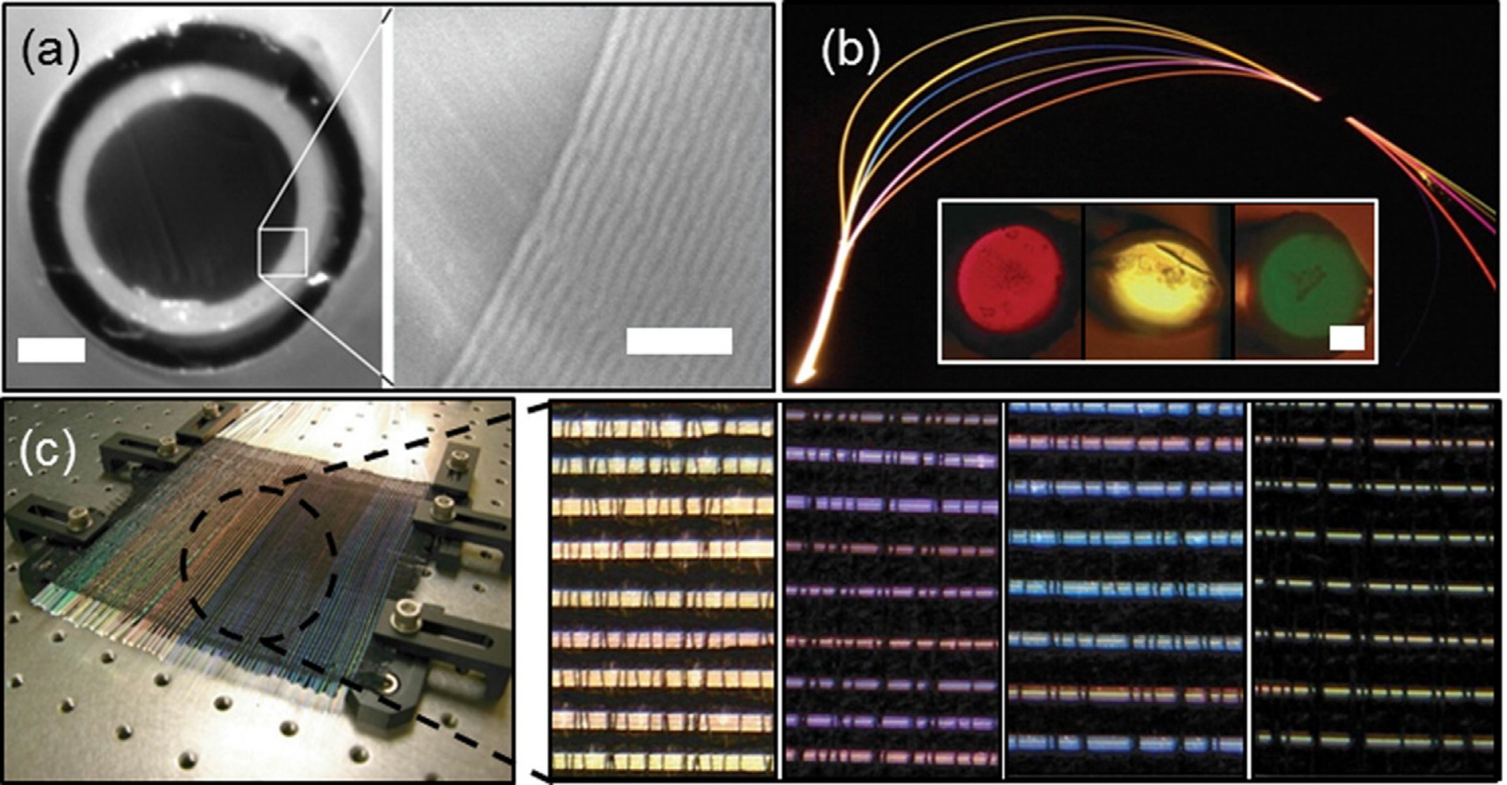
Optic-Fiber and Plasmonic Fibers for textiles (a) Cross section of multilayer structure solid-core Bragg fiber (b) Light scattering phenomena in solid-core Bragg fibers. The different band gap Bragg fibers are shown in the inset. (c) A black silk textile made of Bragg fibers. The various colors of the fibers can be tuned by blending the emitted color and the diffracted color from ambient illumination. Reproduced with permission from Ref. [3]; Copyright 2016 American Chemical Society.

Photonic band gap fibers are a type of Bragg fibers. These textile fibers have a hollow or solid core which is surrounded by dielectric nanolayers arranged periodically according to the varying refractive indices (Fig. 3a) [111]. The preparation of hollow-core Bragg fiber preforms have been done using two methods [135]. One method involves the deposition of two different polymer layers consecutively by solvent evaporation inside a rotating polymer cladding tube.

In second method, the two different polymer films are rolled together inside a plastic tube. Bragg fiber preforms containing a solid-core was developed by rolling together various polymer layers around a rod [130]. PVDF (polyvinylidene fluoride)/polycarbonate or PMMA/PS have been exploited to design Bragg reflectors. Bragg fibers have the property of propagating light by the band gap effect [136]. Band gaps of Bragg fibers are defined as the spectral regions of high diffraction caused due to the periodic multilayer interference effects constitute the band gap of the Bragg fibers (Fig. 3b inset). The spectral position of the band gap is influenced by the refractive index of the core and geometry of the multilayer. Thus, a spectral filtering application can be realized through a band gap guidance mechanism [137]. This property can find applications in textiles for optical sensing [111], [138], and photonics [128]. The Bragg fiber can selectively propagate a particular range of wavelengths while all the other colours are scattered out of the fiber. Thus, it enables to tune the colour of the fiber [139].

A solid-core Bragg fiber hand woven on a Dobby loom was used as a photonic textile [140]. (Fig. 3c). This fabric showed various repetitive colored bands in different colouration. Colouration could be also achieved in cotton fabrics by applying arrays of plasmonic metal nanoparticles such as Ru, Au and Ag [141]. The close packing of the nanoparticles decorated on a garment can produce various colours in the fabrics depending upon plasmon resonance.

## Sensors on textile

**Fig. 4:**
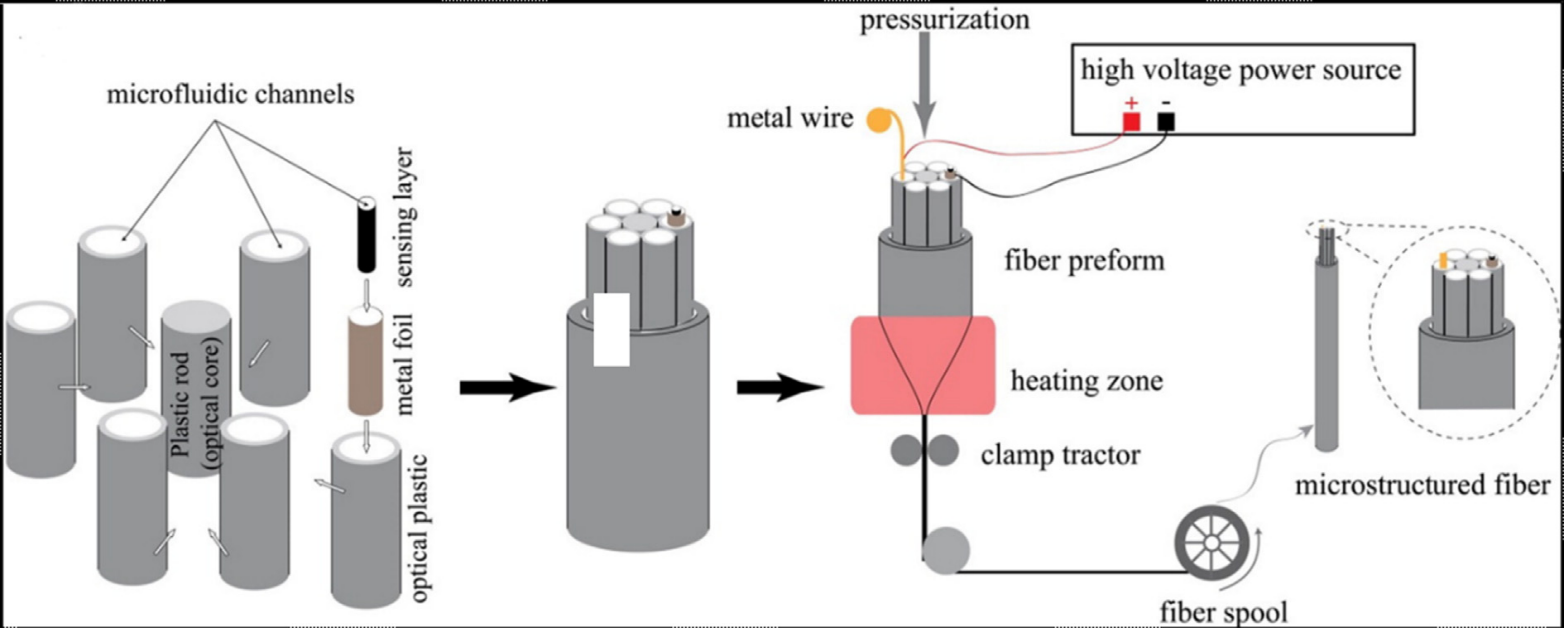
Schematic development of multifunctional nanofibers for sensing applications; Adapted with permission from Ref. [3]. Copyright 2016 American Chemical Society.

Various kinds of sensors can be integrated on the textile for a variety of applications; such as, heat sensors, touch sensors, pressure sensors, optical sensors, chemical sensors, olfactory sensors etc. [142]. Carbon-based nano-materials such as carbon nanofibers, graphene and carbon nanotubes (CNT) have been broadly examined for use as light weight, flexible, and high strain sensors, which may be used in the fields of smart garments, health monitoring, and human motion detection [143], [144], [145]. Carbon-based nanoparticles have been produced using different techniques and are homogeneously dispersed within polymers for application as strain sensors. Strain sensors were formed through direct film-casting and electrospinning techniques [146]. Carbon-based nanofibers, and their woven materials have been analyzed for use in efficient performance strain sensors. Strain sensors have also been developed using human hairs coated with graphene [147]. Following spray coating, carbonization and stabilization; silk and cotton fabrics were also used for strain sensors [148], [149]. Currently, plasmon based sensors have found wide applications for smart textiles. Plasmonic sensors have been found to exhibit high sensitivities for biochemical sensing. Various plasmonic optical fiber sensors can be developed using the drawing methods [150]. A plasmonic fiber sensor works on a plasmon resonance principle. A surface plasmon mode situated on a metal/dielectric interface is excited by an optical fiber core-guided mode due to resonance; when the phase-matching condition arises between the two modes at a certain frequency. The changes in the refractive index of a material on the metal layer alters the phase-matching condition, thus spectral dip at resonance is displaced which is recorded as a signal. Apart from the use of conventional single- or multimode optic fibers for the design of a plasmonic sensor, various modifications are followed viz; etching, cladding or polishing and subsequent further deposition of several tens of metal nanolayers [151]. These series of methods pose various challenges for the development of plasmonic fiber sensors, however, employing stack-and-draw technique can ensure fabrication of a good plasmonic fiber sensor (Fig. 4). Touch sensor fabrics have also been fabricated employing flexible capacitors in the fiber [113]. The capacitor fibers were weaved into a 1D sensor array using a Dobby loom and was then incorporated into a wool matrix. Fifteen capacitor fibers were employed to design the touch sensor fabric. These capacitor fiber when touched with a finger, there occurs change in the voltage distribution and local current which is recorded to sense the touch. These fiber capacitors can also be integrated with other conductive fibers or battery fibers so as to design a functional electric circuit on the garment. This feature can find applications in programmable textiles, safety clothes, and fashion. Fabrics sensitive to pressure have also been developed [152]. For pressure sensors, the fibers were coated with organic conductive polymers such as poly(3,4-ethylenedioxythiophene) and poly(styrenesulfonate) along with a dielectric perfluoropolymer film, using a dye-coating method. These processed fibers were woven as wefts and warps, and the pristine nylon fibers were used to fill the rest of matrix. The nodes where the fibers intersected formed the capacitors. When the fabric was imposed 4.9 N-cm^−2^ pressure, its capacitance changed from 0.22 pF to 0.63 pF possessing a sensitivity range of 0.98–9.80 N-cm^−2^ [152]. Similarly, the temperature and humidity sensors are also incorporated in the fabrics [153]. Advanced techniques like photolithography and inkjet printing has been employed to make the sensors woven into fabrics.

The capacitive humidity and resistive temperature sensors were based on flexible polymer foil substrate and then incorporated into fabrics [154]. To develop such sensors, metal films were also deposited on polyimide sheet substrates. The photolithography made sensors are generally covered by a photoresist film on the substrate. For humidity sensors, cellulose acetate butyrate is used as a sensing medium and is spray-coated on the capacitor through a stencil mask. In inkjet printing, the cellulose acetate butyrate in solubilized in hexyl acetate and is printed on the substrate to get a 5 μm thick film over it. The sensing device is covered by a gas-permeable hydrophobic membrane. Subsequently, such sensors are weaved into the fabrics using a machine in the weft direction with a twill (1/8) pattern. The temperature and humidity sensors have been introduced in the textile along the weft direction so as to replace the weft yarn.

Conductive yarns are now used in place of warp threads so as ensure contacts between the sensors inside a textile fabric. The temperature sensors can be used to sense in the range from 10 to 80 °C with a 5 °C sensitivity. Humidity sensors have 10% sensitivity and are operational in the range from 25 to 85% [154]. The textiles with temperature and humidity sensors can also have LEDs to give optical sensing response [155]. There are various other sensors in textile which sense change in capacitance, inductance and resistance. These textiles are installed with very small chips functioning as analog-to-digital converters, multimeters or amplifiers. Metal − organic frameworks (MOFs) when integrated with quantum nanorods and incorporated in a cotton fabric can act as efficient colorimetric sensors for sensing of toxic gases [156]. A Cu benzene tricarboxylic acid MOF-199 was used in a fabric designed by Matilda Ceesay which could control and capture the release of an insecticide called permethrin. This fabric could find applications in mosquito repellency in malaria dominant areas [157].

## Harvesting human energy for electronic applications through textiles

The human body motions, generation of body heat and fluidic pressures are the very good sources of renewable energy [158], [159]. The sunshine may also contribute to the the overall energy reservoir of the human body. The biomechanical motions and body heat contribute approx. 4.8 W [160] and 67 W [161], [162], [163], respectively; whereas the fluidic pressure may contribute upto100 W [164], [165]. The solar energy density of approx. 100 mWcm^−2^ is also a rich source of energy that a human body can receive [166], [167]. A human body fully installed with all the smart electronics may require energy ranging from 200 μW to 1 W [168]. It is believed that the whole energy requirement can be met by harnessing the human body associated energies. In recent years, various wearable devices have been fabricated to harvest all these energies [169], [170]. The most promising ones include piezoelectric nanogenerators (PENGs) [171], [172], [173], triboelectric nanogenerators (TENGs) [174], [175], thermoelectric generators (TEGs) [176], [177], solar cells (SCs) [178], [179], [180], biofuel cells (BFCs) [181], [182], and hybrid generators (HGs) [183], [184]. All these working mechanisms have some limitations such as bulkiness, larger weight, rigidness etc. which make discomfort to the wearer [185]. Also, the polymer thin films used in these harvesting devices have poor breathability and flexibility [186], [187]. So, the development of this technology without compromising the comfort of the wearer is still a challenge. Based on the various energy sources in the human body, the different energy harvesting mechanisms can be outlined as below.

### Biomechanical energy harvesting in textiles

Various biomechanical movements in human body viz; limb movements, breathing, blood flow and organ movements contribute the energy reservoir of human body [188]. The integration of smart textile technology for harvesting of these energies can provide a convenient and less costly energy backup for on-body electronics. The various principles behind mechanical energy harvesting involves the electrostatic effect [189], electromagnetic effect [190], piezoelectric effect [191], [192] and tribo-electrification [193], [194], [195]. The integration of biomechanical energy harvesters with textiles have some critical concerns associated with it. Firstly, the devices must be very sensitive to the various mechanical effects so that good response is achieved. Second, the comfort and breathability of the textile must be maintained. Further, the rigid structure of the magnets and coils that bring in the electromagnetic transduction brings limitation on the fabrication of wearable textile. [196] Contrarily, the piezoelectric effect and triboelectric effect based nanogenerators can be integrated with ease to the fabric due to their low weight and flexible characteristics [197], [198].

### Textile based TENGs

**Fig. 5:**
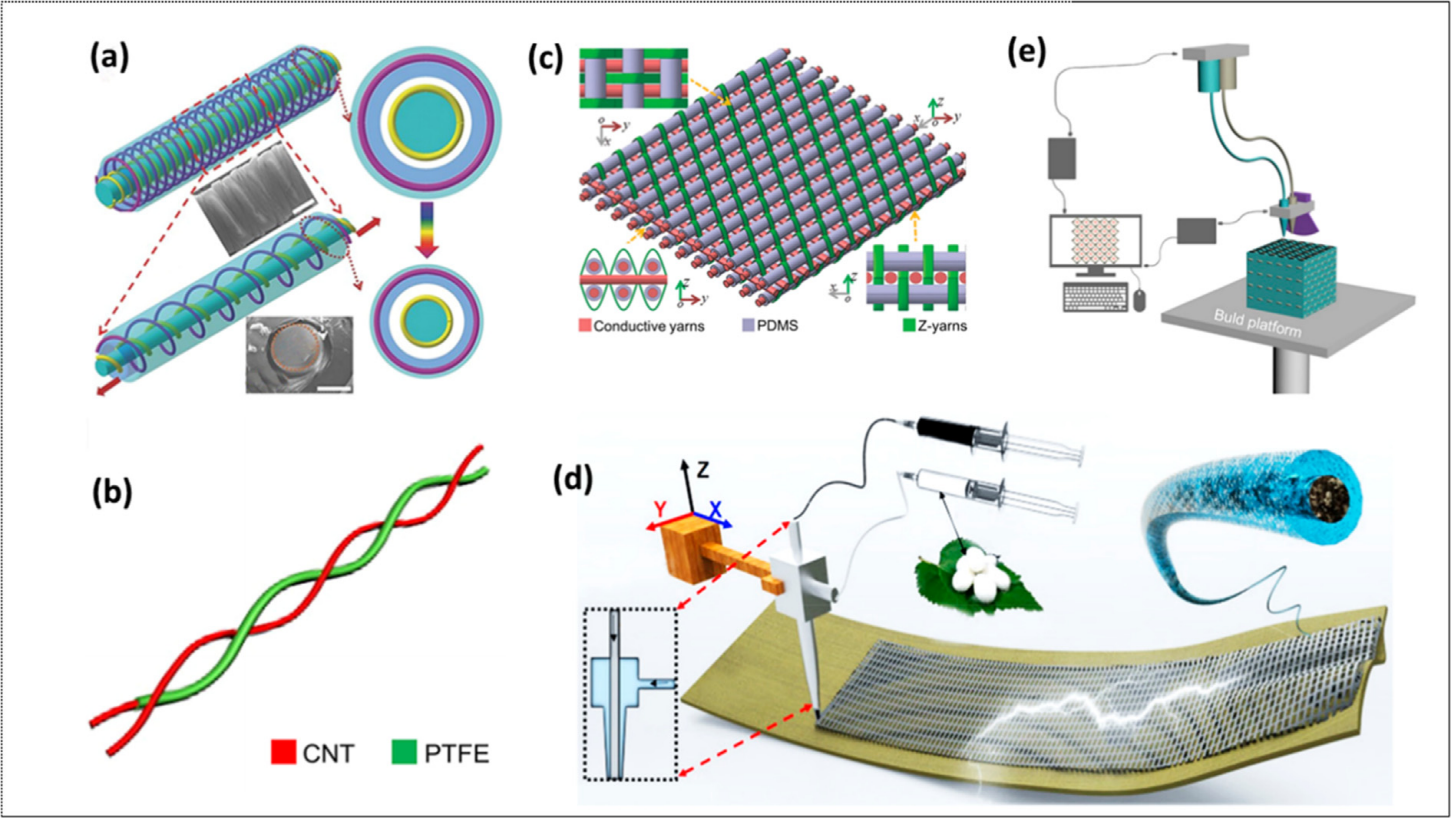
Textile TENGs for harvesting biomechanical energy (a) Schematic illustration of a coaxial yarn-shaped TENG based on yarn intersection; Adapted with permission from Ref. [208] Copyright 2018 WILEY-VCH Verlag GmbH & Co. KGaA, Weinheim(b) Schematic illustration of a pretwisted yarn-shaped TENG based on yarn intersection; Adapted with permission from Ref. [209] Copyright 2014 American Chemical Society (c) Schematic illustration of a 3D orthogonal woven textile TENG based on 3D interlacing or 3D printing; Adapted with permission from Ref. [210] Copyright 2017 WILEY-VCH Verlag GmbH & Co. KGaA, Weinheim (d) Schematic illustration of a hybrid 3D printing system and ultraflexible 3D TENG. Adapted with permission from Ref. [211] Copyright 2019 Elsevier Ltd. (e) Schematic illustration of the 3D printing process and a coaxial fiber-shaped TENG. Reproduced with permission from ref. [212]; Copyright 2018 Elsevier Ltd.

The physical contact between the surfaces of two dissimilar materials produce electrostatic charges [199], [200]. A perturbation imposed by some external mechanical force can generate an electric potential between the two charged surfaces which may produce a polarization current. This polarization induced current is responsible for the operation of triboelectric nanogenerators [201]. Hu and Zheng [202] reported textile-based tribo-electric nano-generators (TENGs), a self-powered sensor and mechanical harvester for wearable process. They analyzed the effect of textile processing methods i.e; weaving, knitting and sewing on the structure pattern and TENG’s efficiency in wash and tailor-ability. They found various material selections suitable for TENGs and surface alteration of conductive textiles lead to generate efficient triboelectricity. From last one decade, TENGs have been used as sustainable power sources in textile to run electric devices [203], [204] or sensors [205], [206]. The integration of TENGs with textile for biomechanical energy harvesting involves three fabrication methods. These methods are layer stacking, yarn intersection, and 3D printing. The layer stacking has further different modes of execution such as Single electrode mode, Contact separation mode and Free-standing mode (Fig. 5) [207].

### Textile based PENGs

**Fig. 6:**
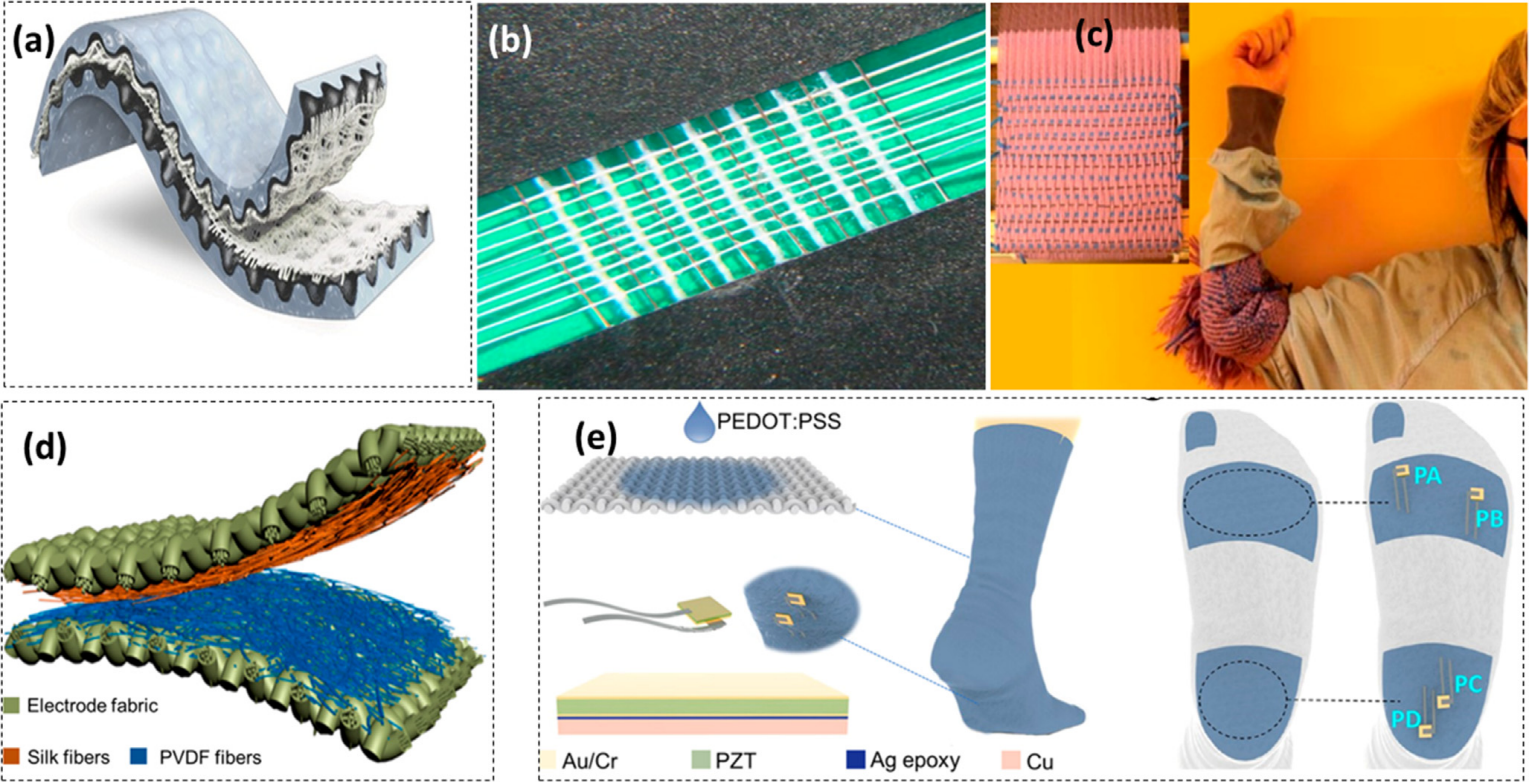
Textile PENGs based on yarn intersection for biomechanical energy harvesting. (a)) Stretchable nano-fiber PENG with a stacked nanofiber mat and graphite electrodes; Reproduced with permission from ref. [218] Copyright 2018 WILEY-VCH Verlag GmbH & Co. KGaA, Weinheim (b) Photograph of a 2D textile PENG by intersecting three kinds of yarns Adapted with permission from Ref. [216] Copyright 2015 Elsevier Ltd. (c) Photograph of a textile PENG mixed weaving with cotton to form an energy elbow pad; Adapted with permission from Ref. [217] Copyright 2017 American Chemical Society (d) Schematic illustration of an all-fiber textile TPENG; Adapted with permission from Ref. [219] Copyright 2018 Elsevier Ltd. (e) Schematic illustration of a cotton sock using the piezoelectric and triboelectric hybrid mechanism; Embedded PZT force sensors labeled as “PA”, “PB”, “PC”, and “PD”. Reproduced with permission from ref. [224]; Copyright 2019 American Chemical Society.

Piezoelectric effect is a working mechanism that involves the application of pressure on a surface. This effect can be integrated with textiles for harvesting of human body associated energies. In this working mechanism, the application of pressure alters the charge distribution which eventually produces an internal electrical field [213]. Thus, the mechanical motions of the human body can translate into the generation of electricity [214]. The various materials used as the piezoelectric materials since the piezoelectric effect was introduced in 1880, are polyvinylidene fluoride based materials and metal oxides like ZnO, lead zirconate titanate (Pb [Zr_X_Ti_1−X_]O_3_), and BaTiO_3_. Wang and co-workers (2006) used ZnO nanowires for the first time to fabricate a PENG for energy generation from various small ambient mechanical body movements [215]. Zhang and co-workers (2015) [216] developed PENG from hybrid piezoelectric fiber using aligned BaTiO_3_ nanowires and PVC polymer along with Cu wire and cotton fibers. While integrating this on the elbow pad, they could achieve the output voltage and current of 1.9 V and 24 nA, respectively which is enough to power an LCD. Lu and co-workers (2017) fabricated kilometer-long piezoelectric micro/nanofibers. They exhibited nice electrical and mechanical stability in a cyclic bend–release test. An output voltage of 6 V was obtained on moderate bending. The authors claim that the better efficiency is due to the proximity of conducting electrodes sandwiching the piezoelectric composite layers. Also, the spiral structure enhances the active surface area which increases the output voltage and results in 10–100 times better power efficiency over the earlier reported piezoelectric cables [217]. Siddiqui and co-workers (2018) reported stretchable piezoelectric nanogenerators (SPENGs) based on BaTiO_3_ nanoparticles embedded in polyurethane and poly(vinylidene fluoride-trifluoroethylene) nanofibers. They achieved 40% stretchability, and high stability upto 9000 stretching cycles. This nanofiber SPENG exhibited open circuit voltage (V_oc_) of 9.3 V and short circuit current (I_sc_) of 189 nA [218]. Guo and co-workers (2018) reported an all-fiber hybrid PENG developed by electrospinning silk fibroin and PVDF nanofibers on conductive fabrics. These PENGs exhibit outstanding power density of 310 µWcm^−2^ and are flexible and air permeable to suit the wearability [219]. Qi and co-workers (2020) [220] analyzed the modest and cost-effective approach to synthesize extremely delicate woven wearable textile pressure sensors. The most significant properties of the nano-material lead to increased contact area in stimuli with low external pressure. The as prepared textile pressure sensor based PENG exhibited high sensitivity, wide sensing range, and short response time. Thus PENGs when integrated with textile have a great potential for harvesting of ambient mechanical energy [221], [222] for the operation of *on-body electronic sensors* [223]. Owing to the simple structural design and flexibility, PENGs are being associated with textiles at a great pace to realize a wearable energy solution to human body for *on-body electronics*. These devices are generally fabricated through layer stacking and yarn intersection as demonstrated in Fig. 6 [207].

### Human body heat energy harvesting by smart clothes

**Fig. 7:**
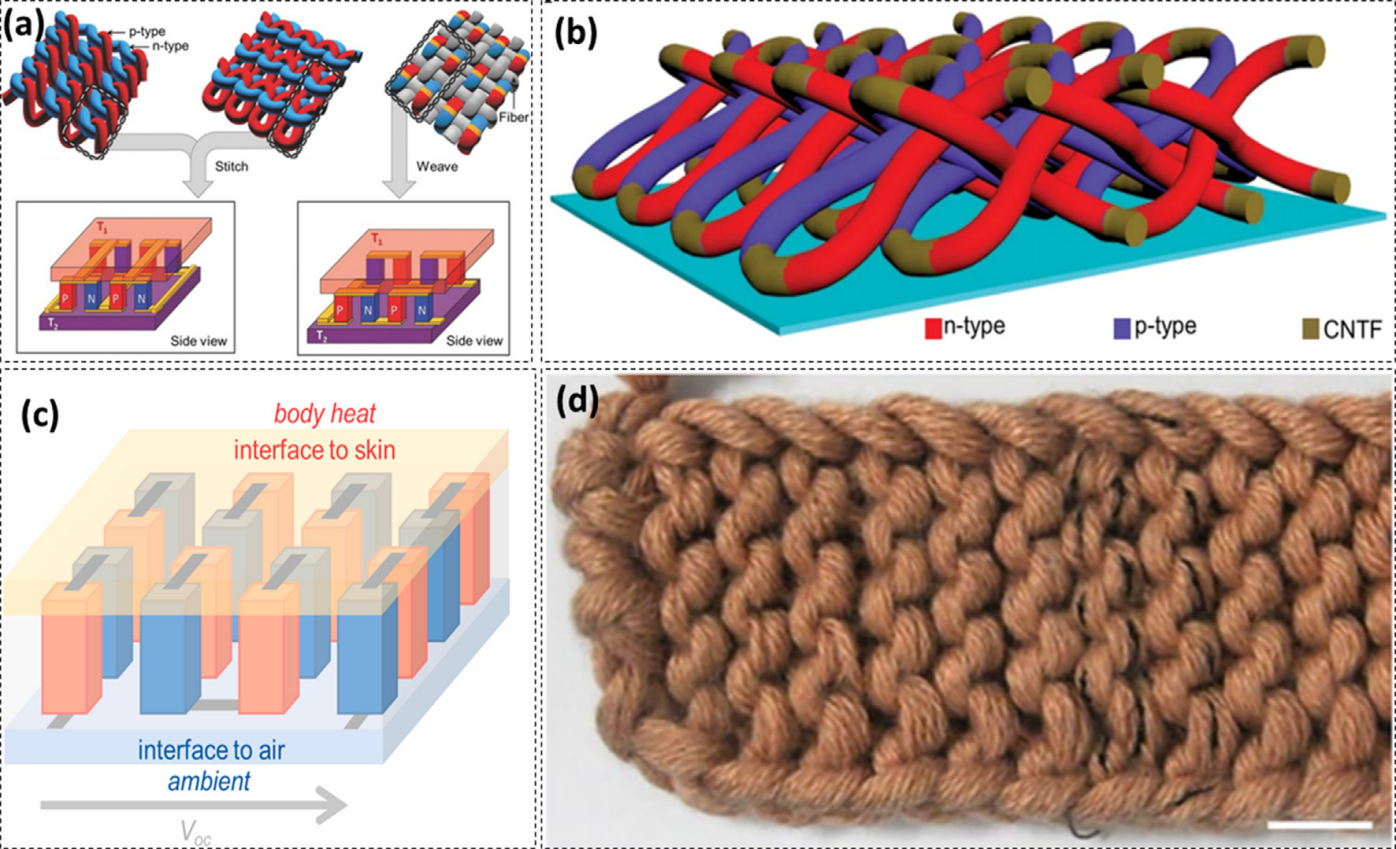
Body heat energy harvesting by Yarn-constructed TEGs (a) Schematic illustration of textile TEGs based on zigzag stitch, garter stitch, and plain weave; Adapted with permission from Ref. [239] Copyright 2016 WILEY-VCH Verlag GmbH & Co. KGaA, Weinheim (b) Schematic illustration of the 3D textile TEG without substrate; Adapted with permission from Ref. [240] Copyright 2020 Nature Publications (c) Schematic illustration of a 3D textile TEG representing a wearable thermopile consisting of several thermocouples connected electrically in series and thermally in parallels; Adapted with permission from Ref. [241] Copyright 2020 Elsevier Ltd. (d) Photograph of the 3D textile TEG without substrate (1 cm Scale bar). Adapted with permission from Ref. [240] Copyright 2020 Nature Publications.

Body heat is a constant source of energy originating from the various metabolic processes, irrespective of the physical activities of a person [225]. The average energy released by a human body is 100–525 W [226]. Harvesting this energy by using smart textiles can be a promising method to feed on-body electronics. Two very important working mechanisms used to harvest body heat energy are the pyroelectric effect [227], [228] and thermoelectric effect [229], [230], [231]. The pyroelectric effect is based on the temperature differences with respect to time whereas the thermoelectric effect is based on temperature differences with respect to space. However, the pyroelectric effect is less efficient as the average human body temperature exhibits a minimal variation with respect to time [232]. The thermoelectric effect arising due to the spatial temperature difference can contantly generate energy of ∼10 mW cm^−2^ [233]. Integrating a thermoelectric generator (TEG) on the fabric, the body heat can transfer charge from body to the generator (Fig. 7) [207]. TEGs have found a great potential for wearable electricity generation owing to the development of highly efficient thermoelectric materials. Textile integrated TEGs with a high efficiency [234], flexibility [235], [236], stability [237], and light weight [238] have been developed so far. The integration of TEGs on the textile generally depends on the textile substrate and the yarns as building blocks. The TEGs too have the limitations as the body-TEG interface is yet to be made highly efficient.

### Biochemical energy harvesting in human clothes

**Fig. 8:**
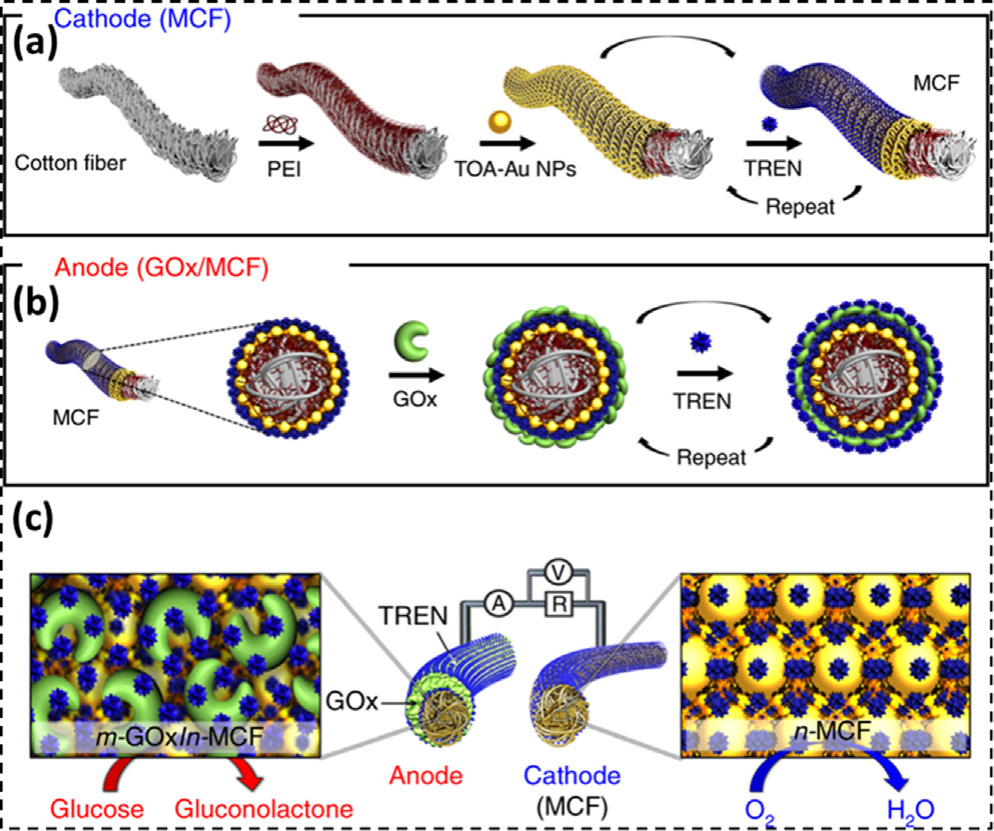
Metallic cotton fiber electrode-based biofuel cell. (a) Preparation of the metallic cotton fiber based cathode and the glucose oxidase-metallic cotton fiber-based anode using small-molecule ligand-induced layer-by-layer assembly. (b) Redox process for an metallic cotton fiber-biofuel cell composed of a cathode and an anode. (c) Demonstration of metallic cotton fiber based biofuel cell performing different reactions at the cathode and anode; Adapted with permission from Ref. [251] Copyright 2018 Nature Publishing.

The biochemical energy is also important source of energy in our body which involves many body fluids, including blood, tears, saliva and sweat [242], [243]. These biochemical forms are considered as renewable and eco-friendly sources of energy [244]. This biochemical energy is stored in the form of glucose, fructose, and lactate and it can contribute up to 100 W in a healthy human body [165]. These biochemicals can be exploited as rich biofuels in a biofuel cell (BFC) [207]. In a BFC, the biofuels get oxidized by the biocatalysts at the anode, release electrons which transfer to the cathode through an external circuit. [245] The electrons at the cathode reduce oxygen to produce electricity. The two most celebrated fuel cells in this regard are the enzyme biofuel cell (EBFC) [246] and the microbial biofuel cell (MBFC) [247]. The EBFC is considered better in the sense as it has higher biocompatibility [248], conversion efficiency [249], and can be easily miniaturized [250]. For the fabrication of a EBFC device on a fabric, smart textiles are being used as the enzyme supports where as a yarn is designed to fabricate fiber electrodes. Kwon and co-workers (2018) [251] developed a BFC from porous metallic cotton fiber by making layer by layer assembly of the active catalysts using small-molecule linkers. It was believed that these systems significantly enhance the direct electron transfer rate between the conductive supports and enzymes (Fig. 8). They used the Au nanoparticles owing to its many benefits including high conductivity and biocompatibility. The metallic cotton fibers acts as the conductive substrate for the deposition of the anodic enzymes. They also act as electrocatalytic cathode for the ORR reaction. The researchers tuned the amount of Au nanoparticles in the cotton fiber so as to achieve a 3D porous structure which could offer enhanced conductivity and ORR activity without using cathodic enzymes.

### Solar energy harvesting by textiles

**Fig. 9:**
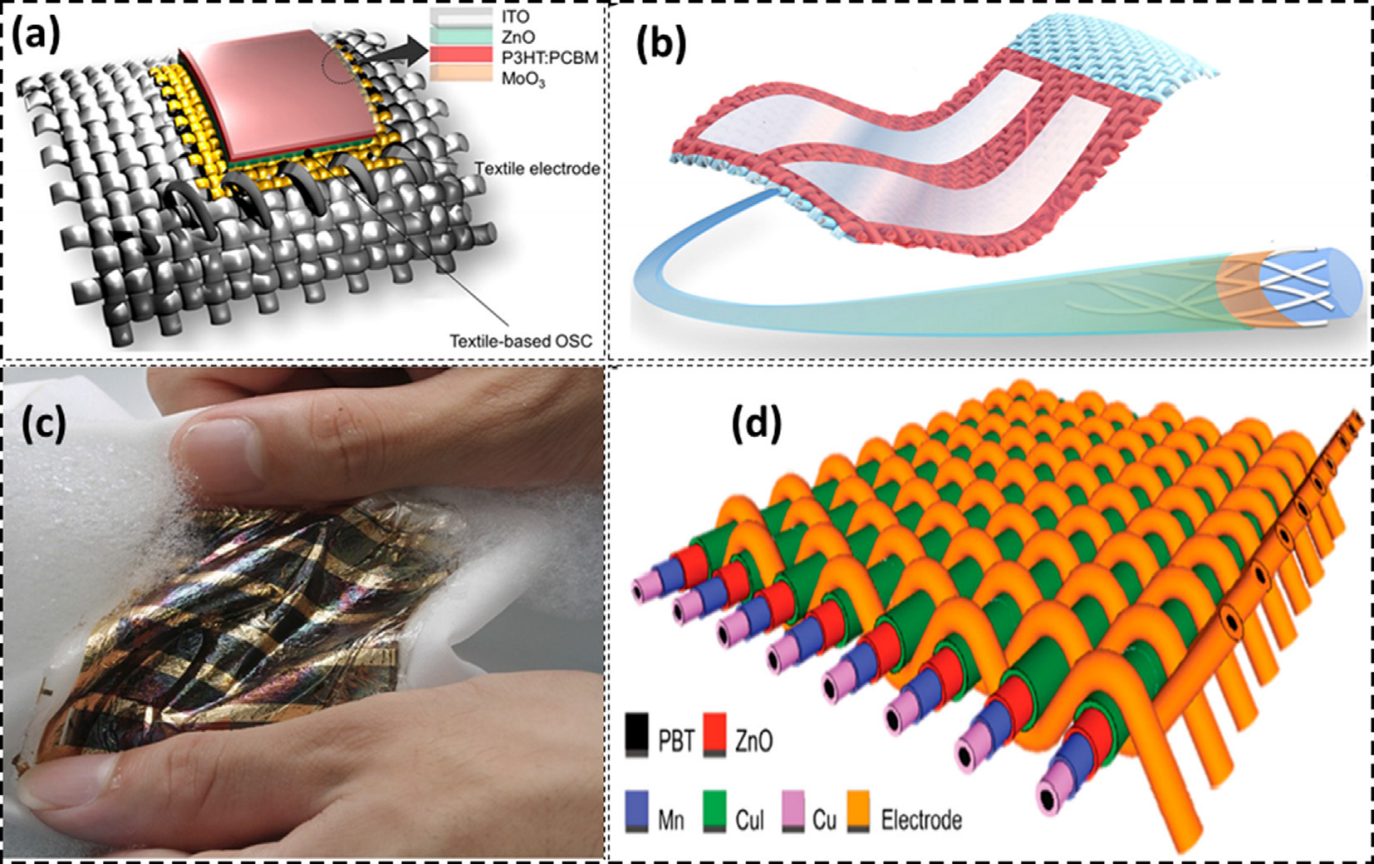
Textile Solar Cells made by layer stacking (a) Schematic illustration of a stitchable textile Organic solar cell; Adapted with permission from Ref. [267] Copyright 2014 Elsevier Ltd. (b) Schematic illustration of a textile organic solar cell built on a polyester fiber-based substrate; Adapted with permission from Ref. [268] Copyright 2017 Elsevier Ltd. (c) A washable textile organic solar cell; Taken from Ref. [180] Copyright 2017 Nature Publishing. (d) Schematic illustration of a solid-state textile DSSC by yarn intersection; Adapted with permission from Ref. [269] Copyright 2016 WILEY-VCH Verlag GmbH & Co. KGaA, Weinheim.

The solar energy which makes about 100 mWcm^−2^ is considered to power on-body electronics [252]. The annual capacity of solar energy globally is 1575-49837 exajoules (EJ), which is almost three times higher than the total global consumption of 600 EJ [253]. Various kinds of inorganic semiconductor metal derivatives and their hybrid nanocomposites have been exploited to harness solar energy for various functions such as self-cleaning and energy generation [254], [255], [256], [257], [258]. Harifi and co-workers [259] developed lightweight, flexible and highly durable polyester fabric using TiO_2_/Fe_3_O_4_/Ag nano-photocatalysts for photo-transformation. They found that wettability is required for the photocatalytic reaction in the fibrous materials. They concluded that nano-photocatalysts mounted on the textile fabrics result in the photocatalytic conversion of acetic acid to solar fuel. The solar energy can be better harnessed by the photovoltaic effect using highly efficient solar cells. In these solar cells the current is produced by the photoexcitation of the active layers to produce the electrons and holes. These solar cells have been divided into three main generations [260], [261]. Initially a wafer-based solar cell which involved a crystalline silicon was used. Later on, thin film based solar cells were produced using cadmium telluride, amorphous silicon, and copper indium gallium selenide etc. Hatamvand and co-workers (2020) [262] reviewed planar and fiber shaped solar cells. The various limitations and challenges to be encountered for the latest technology development were discussed. They concluded that synchronization of wearable properties and development of planar-shaped solar cells (PSSCs) on the textile fiber is the main challenge. However, organic material based solar cells such as dye-sensitized solar cells (DSSCs), perovskite solar cells (PSCs), etc. are now being widely explored and applied for smart textile technology. The organic solar cells [263], PSCs [264], [265] and DSSCs [266] have found a potential application for powering on-body electronics owing to light weight, flexibility, easy fabrication, abundance and low cost. For fabrication of textile solar cells two techniques are being generally employed i.e, layer stacking and yarn intersection as demonstrated in Fig. 9 [207].

### Hybrid energy harvesting by textiles

As the energy requirements for the on-body smart textiles is increasing every year, the energy from a single source falls short of the requirements. Also, it is often improbable to use all the energy forms from the human body. For example, on a cloudy day or during night, the solar energy backed mechanisms can’t work. Hence, researchers have developed hybrid generators on textile which could harness energy from more than one sources so that the increasing demands can be met [207], [287]. Say for example, a person walks on a hot sunny day, he involves the biomechanical energy, the solar energy, the body heat, and also the biochemical energy from perspiration. Hence, for efficient harnessing of these energy forms simultaneously requires a hybrid generator so that a optimized power supply could be provided to smart textiles. However, these hybrid generators are not capable to harvest three or more energy forms simultaneously with a satisfying efficiency due to the complicated structural limitations. Their integration with the textiles is being seen as a promising research prospect in future so as to develop a sustainable power source for on-body electronics.

## Environmental and health concerns associated with smart textiles

The extensive use of nanoparticles and nanomaterials for the production of smart textile raises concerns and may not be completely beneficial. Various toxic chemicals are used in their production and nanoparticles can leach from the final products and find their way into the water sources after washing of the textiles. To illustrate the problem, a significant amount of Ag nanoparticles have been observed to wash into the waters from a silver treated blanket. Measurement showed that the blanket loaded at 109.8 ± 4.1 mg Ag kg^−1^ could lose almost 4.8 ± 0.3 mg Ag kg^−1^ into a user’s sweat over the course of 1 h use [288]. Commercial socks containing nanoparticles with concentration 1360 μg Ag g^−1^ leached upto 650 μg of Ag into 500 ml of distilled water within 24 h [289]. The extent of leaching was found to depend on the concentration of the Ag nanoparticles in the fabric and also on the pH of water or sweat. Another analysis showed that a fabric containing TiO_2_ nanoparticles at levels ranging from 2.9 to 8.5 g Ti kg^−1^ could leach TiO_2_ at amounts dependent on different pH [290]. Acidic sweat leached 63 ± 13 μg g^−1^ L^−1^ , whereas, 38 ± 13 μg g^−1^ L^−1^ was found in the alkaline pH [291]. Ag − chloro complexes were detected where the sweat contained high concentrations of chloride ion. Ag nanoparticles are known to be hazardous to aquatic biota including fish and plankton [292]. The antimicrobial nature of Ag nanoparticles may also disrupt the microbial habitat in sewage treatment plants [293]. Solid nanoparticles also pose concerns in the workplace as they may get inhaled and get into the bloodstream [294].

It is clear that much more research is required to fully understand these concerns. Garments manufactured under different conditions may have different stabilities and durability and so lose material at different rates. Considering the severity of these assessments, people need to be much more aware of the influence of toxic nanomaterials on the environment. Manufactures need to ensure that their nanomaterial based textiles are highly durable. At the same time, the general public needs to be educated regarding the proper washing methods and encouraged to use low temperature, low agitation washing with an appropriate organic detergent and to avoid tumble-drying. These measures may mitigate the environmental impact. Further, recycling the processed textile will decrease the production and release of toxic nanomaterials from disposal. As well as the consumers, since nanomaterial based textiles are becoming a blooming economy, concerns regarding health risks of the workers who manufacture them need to be addressed. Hence, proper government regulations regarding this industry and market need to be put in place so that these exciting developments can be realized within the limits of environmental safety.

## Future directions

From the above discussion, it is clear that the incorporation of high performance, miniaturized microprocessors in textiles can do wonders in terms of collecting, processing and using information throughout smart garments. These smart textiles promise breakthrough applications in the health, security, and fashion industries. Garments integrated with specialized sensors can monitor the wearer’s physiology and body posture which can draw attention to and correct problems or abnormalities before they become serious. Some areas where advances could be made in this direction include the installation of pressure and posture sensors in shoes to indicate incorrect posture or body-weight. Vibration nanomotors in the garment could enhance the wearer’s blood circulation and can also stimulate weight loss. Wearable capacitors can provide power to all the external or internal devices in a human body. One important application, particularly in hot climates but also in patients suffering from fever, which can be achieved through smart textile is body cooling. For this purpose, very efficient dense fabric batteries or solar cells require to be designed which could promote efficient cooling of the body. Infra-red radiation reflectors can also be useful in this application.

The smart textiles have a great scope in fashion industry and introduction of newer technologies is always welcomed. As discussed earlier, smart textiles have been found used for fashion in terms of luminescence, colours, holography, by the use of plasmonics, photonic crystals, LED displays etc. These garments could be further integrated with sensors to measure temperature, touch, humidity, light intensity or movement. Dynamic graphics can also be realized if wearable fiber optics can be developed to create displays. A fabric developed by Cambridge Consultants called Xel flex fabric which contains optical fiber sensors to detect the movements of the human body [3]. This fabric can find application in sports coaching and physiotherapy. Hence, fully functionalized garments could be realized in the near future which could continuously monitor the wearer’s health, movements and other activities like sports and threats. In this regard, Organic Light Emitting Diodes (OLEDs) have been exploited for formation of thin films. Quantum-dot light-emitting diodes (QLEDs) have also been considered as they have an extra active layer with respect to the OLEDs [295]. These QLEDs are expected to consume lesser energy and could produce larger luminescence. The better optical properties could be integrated with other optical components such as diffraction gratings and lenses to produce mesmerizing effects [296]. Photonic crystals have different dielectric constant and refractive indices along the three orthogonal directions from where band gap fibers can be fabricated. This feature can be explored to design thin band optical effects. Holography has also a great scope for development in the textile industry [281]. Holograms can be projected into helmets or onto glasses for virtual reality applications. Also holographic sensors have been developed on the fabrics to monitor metabolic activity [297]. Metamaterials are extraordinary structures developed by combining nanomaterials in periodic structures at length scales shorter than the desired wavelength [298]. They have negative refractive indeces which may allow the design of ‘cloaking devices’ for making objects invisible as electromagnetic radiation cannot pass through the material. Such devices have been developed at microwave [299] and THz frequencies [300], but invisibility garments in the visible region are yet to be realized. Textiles can be integrated with some specific receptors or biomarkers and fluorescent dyes which can perform rapid and timely physiological diagnostics [301]. In the future, all these applications based on display and sensing characteristics will be operated through smartphones [302]. Flexibility, comfort and breathability are major concerns associated with the integration of smart nanomaterials in textile as without them, garments will not be acceptable to the customer. Researchers are therefore focusing on strategies to maintain these desirable charactersitics while processing the textile. Traditionally, cotton is often considered the best choice due to its smoothness, absorbency and breathability. However, its wide use in fashion technology is limited due to its low strength, easy wrinkling, soiling and flammability [303]. Synthetic counterparts are available without these limitations but they are not as comfortable as cotton. Hence, researchers aim to combine the advantageous features of cotton with those of synthetic fibers [304] to produce nano-engineered functional textiles compromising on the comfort of the clothes [108]. Guan and co-workers are addressing this by fabricating 3D conformal porous microstructured textiles. They demonstrated the influence of solution concentration, temperature, relative humidity, nanomaterials, and fabric substrates on the porous structure, flexibility and durability of the product and claim that their strategy for nanomaterial integration on textile can realise the development of wearble fabrics with high flexibility, comfort and functionality [305].

## Conclusion

The work described in this article shows that the production of smart textile materials has seen tremendous advances in recent years but that there is the potential for even more useful products to be developed. The advances in fabrication methods for nanomaterial based textiles, the potential market demand and subsequent scope for research has attracted many new workers to the area. The last two decades or so has seen the integration into textiles of various nanomaterial based structures such as metallic or metal oxide based nanoparticles, carbon nanotubes, nanoelectronics and optical components including Bragg diffraction gratings. These materials were prepared using various fabrication methods such as spray coating, impregnation, lithography, spray coating, fiber drawing or weaving. To produce effective electronic or optical functionalities, the surfaces of textile fabrics have been modified with nanomaterials in order to produce flexible and wearable garments with high aesthetic appearance so as to be attractive to the consumer. Applications that have been realized by nanotextiles include water repellence, antibacterial properties, UV protection, odor control, wrinkle resistance, durability, and antistatic properties. More advanced applications which are yet to be realized on a large scale involve energy storage, sensing, drug release, optics, electronics and photonics. Along with the bloom of the smart textile industry, environmental concerns are also magnifying. So, life-cycle assessments and the potential toxicity of leached nanomaterials from textiles needs to be critically evaluated. It has been reported that production of textiles and apparel contributes approx. 10% of the total carbon emissions in the environment. Textile dyeing contributes 17–20% to water pollution. The accumulation of nanomaterials in the water bodies due to leaching from textile seems inevitable so that action is needed before their use becomes widespread, in contrast to the way that microplastics were allowed to be released uncontrolled into the environment. Hence, the environmental controls need to be put in place. Awareness in this regard must be inculcated in the general public so that only safe, recyclable and climate neutral nanotextiles are produced.

## Compliance with Ethics Requirements

*This article does not contain any studies with human or animal subjects.*

## CRediT authorship contribution statement

**Mudasir Akbar Shah:** Conceptualization, Methodology. **Bilal Masood Pirzada:** Writing – original draft, Software, Data curation. **Gareth Price:** Supervision. **Abel L. Shibiru:** Visualization, Investigation. **Ahsanulhaq Qurashi:** Supervision, Validation.

## Declaration of Competing Interest

*The authors declare that they have no known competing financial interests or personal relationships that could have appeared to influence the work reported in this paper.*
